# Factors associated with sunbed use among 3692 outpatients in 18 centers of the Italian Cancer League (LILT)

**DOI:** 10.1038/s41598-021-02026-3

**Published:** 2021-11-30

**Authors:** Simona Mastroeni, Francesca Sampogna, Nidia Melo Salcedo, Francesco Ricci, Luca Fania, Flaminia Antonelli, Damiano Abeni, Mario Cristofolini

**Affiliations:** 1 Clinical Epidemiology Unit, IDI-IRCCS, Via dei Monti di Creta 104, 00167 Rome, Italy; 2 Melanoma Unit, IDI-IRCCS, Rome, Italy; 3 Dermatology Department, IDI-IRCCS, Rome, Italy; 4 Dermatology, Università Cattolica del Sacro Cuore, Fondazione Policlinico Universitario A. Gemelli IRCCS, Rome, Italy; 5 Italian Cancer League (LILT), Trento, Italy

**Keywords:** Skin cancer, Risk factors

## Abstract

Indoor tanning is associated with an increased risk of skin cancer. Nonetheless, its use is still widespread. We aimed to investigate the socio-demographic and clinical characteristics of sunbed users in a group of participants in the skin cancer prevention campaign organized by the Italian Cancer League (LILT). During almost 2 years, 4409 individuals were screened in 18 centers. Participants reported having used sunbeds before the age of 15 years in 2.2% of cases, while after age 15 the prevalence of use was 22.2%. Participants with complete information were 3692. Sunbed users aged > 15 years were significantly more frequently females, young, living in Northern Italy, highly educated, and current or former smokers. They had darker phototype, more common nevi, had used sunbeds more frequently before the age of 15, reported a history of sunburns, and use of sunscreens. Indoor tanning is an important public health issue and a relevant target for primary prevention. However, not all countries have adopted the recommendations issued by the World Health Organization (WHO) on health risks associated with artificial tanning. A deeper insight into the topic may contribute to identify the best prevention strategies.

## Introduction

Indoor tanning is a well-established risk factor for skin cancer. Several studies have reported an increased incidence of melanoma and non-melanoma skin cancer following such exposure^1,2^. In particular, it has been shown that the use of sunbeds is more dangerous if started under 25 years of age or prolonged for more than ten sessions. The International Agency for Research on Cancer (IARC) included the use of UV-emitting tanning devices in the list of type 1 carcinogens, thus defining it as “carcinogenic to humans”^3^.

The use of indoor tanning is associated with demographic factors, such as young age and female gender, but also with the country of origin, as a result of cultural and climatic reasons^4^. In the 2015 National Health Interview Survey of U.S. adults, the highest rates of sunbed and sunlamp use were found among non-Hispanic white women between the ages of 18 and 24 years old, with 17.2% of them reporting use of these devices in the past year. Moreover, 3.6% of U.S. women reported having performed more than ten sessions per year^5^. In Northern and Western Europe, indoor tanning is even more widespread, with a lifetime exposure prevalence of 41.6% in adults, closely followed by the U.S. (35.4%), while lower values are recorded in Australia (10.7%). On the basis of the proportional attributable risk for these 3 regions, Wehner et al. estimated nearly half a million new cases of skin cancer diagnosis each year due to indoor tanning^6^.

Studies on this topic usually recruit participants in specialized hospital and/or academic settings, targeting a specific population by age or the general population^7^. In the present study we aimed to collect information on people who underwent voluntary screening for skin cancer, to investigate the socio-demographic and clinical characteristics of sunbeds users in a group of participants in the skin cancer prevention campaign organized by the Italian Cancer League(LILT).

## Methods

All persons who participated voluntarily in the LILT campaign on the early diagnosis of skin cancers were invited to join the study. The campaign took place from February 2018 to November 2019 and involved 18 regional LILT Italian centers. During the skin examination, the dermatologists, using a standardized data-collection form, registered information on sociodemographic characteristics, skin and phenotypic characteristic, presence of pigmented lesion (common nevi, freckles, solar lentigo, actinic keratosis), atypical nevi and suspicious skin lesions, medical and family history, lifestyle and sun exposure behaviors, use of sunscreen and sunbed. Questionnaires, stripped of personal identifiers, were sent by mail or electronically to the Clinical Epidemiology Unit of IDI-IRCCS and data were entered in a unique database developed with EpiInfoTM version 7.2. The study was approved by the Institutional Review Board of LILT, Rome, Italy. Informed consent was obtained from all subjects. The study was performed in accordance with the Declaration of Helsinki.

Age was categorized as follows: < 35, 35–54 and 55 years and higher; education was categorized into years of school attendance: ≤ 8 (primary to secondary school), 9–13 (high/professional school), > 13 (university degree and higher) and then dichotomised into low (≤ 8 years) vs. high (≥ 9 years); Fitzpatrick’s skin phototypes were categorized into I–II (“fair”) and III–VI (“dark”); hair colour into black, dark brown, light brown, blonde/red; eye colour into dark brown/black, light brown, blue/green; presence of freckles, solar lentigo and actinic keratosis as “no”/ “yes”; number of common nevi in the whole body into < 10, 10–30, 31–50, 51–100 and > 100; sunbed use was categorized as “never” and “ever used”; history of sunburns before and after the age of 15 and use of sunscreen were categorized as “no”/ “yes”; smoking habits was classified as “never smoker”, “current smoker” and “former smoker”. Participants were defined “at risk” if at least one condition was present among > 40 common nevi, atypical nevi, first degree family or personal history of melanoma or non-melanoma skin cancer, presence of outcomes from solar damage (i.e. sunburns, solar lentigo, etc.) or in the presence of suspicious skin lesions. The cutoff of 40 nevi was chosen on the basis of the meta-analysis by Gandini et al.^8^ where it was shown that individuals with 41–60 nevi had more than a two-fold risk of melanoma compared to individuals with no nevi. Participants defined “at risk”, the presence of the single factors that contribute to their categorization as “at risk”, the use of sunscreen, the presence of atypical skin lesions, and the need for surgical excision were categorized as “no”/ “yes”.

Groups were compared using the χ^2^ statistics for categorical variables, and the Mann–Whitney U test for continuous variables. Multivariable logistic regression analysis was performed to examine factors potentially associated with sunbed use after the age of 15 years, while controlling for the main variables of interest. The likelihood ratio tests and Akaike information criterion (AIC) were used in the stepwise selection of all abovementioned variables. The test for trend was used where appropriate. Due to missing data, a complete-case analysis (i.e. “list-wise deletion”) was also performed. The Spearman’s coefficient was used to estimate correlation between the prevalence of sunbed use in each LILT local centre and GPS latitude and longitude, mean annual of minimum and maximum temperatures, mean number of days with sun, with frost, without precipitation and number of consecutive days without precipitation, respectively. The Istat Meteo-climatic and hydrological data survey (https://www.istat.it/it/archivio/251803, last accessed on January 15th, 2021) was used to obtain information on meteorological data. Statistical analyses were carried out using the Stata Statistical Software: Release 15. College Station, TX: StataCorp LLC.

## Results

Overall, 4409 individuals were screened during the LILT campaign. Of these, 2906 (65.9%) were enrolled in ten LILT outpatient clinics from Northern Italy; 490 (11.1%) were enrolled in two LILT centres from the Central Italy, and 1013 (23.0%) from six LILT centres from Southern Italy. Data on gender was reported on 4309 participants (97.8%) of whom 63.0% were females, with an overall female/male ratio of 1.7 (ranging from 1.3 to 3.0 in the different centers). Age was recorded for 87.9% of participants, with a median age of 47 years (Interquartile Range (IQR) = 34–59). Information about education was available in 77.4% of cases, 83.7% of whom attained high education (≥ 9 years). Skin phototype was reported for 81.5% of participants, and of them 2775 (77.4%) had dark skin phototypes (III–VI). Participants with information on the use of sunbeds both before and after the age of 15 were 3692, and statistical analysis has been restricted to them. Participants reported having used sunbeds before the age of 15 years in 2.2% of cases, while after age 15 the prevalence of use was 22.2%.

Sunbed users aged 15 years or higher (Table 1) were significantly more frequently females, young, living in Northern Italy, highly educated, and current or former smokers. Also, participants who reported using sunbeds had lighter hair colour and darker phototype, had more common nevi and fewer actinic keratoses, had used sunbeds more frequently before age 15, reported a history of sunburns, and the use of sunscreens. Among the characteristics that defined subjects “at risk”, common nevi > 40 and outcomes from solar damage were associated with sunbeds use.

**Table 1:** Description of the study population and prevalence of sunbeds use according to sociodemographic and clinical characteristics, habits and risk factors.

	All (N = 3692) N^a^ (%)	Use of sunbeds: yes (N = 660) N^a^ (%)	Prevalence of sunbeds use (%)	*P*value^b^
Overall	3692	660	17.8	
**Age, years**				
Mean (SD)	46.8 (16.5)	45.1 (12.1)		
Median (IQR)	47 (34–60)	44 (36–53)		0.001^c^
< 35	860 (26.3)	128 (20.2)	14.9	
35–54.9	1327 (40.6)	383 (60.3)	28.9	
≥ 55	1083 (33.1)	124 (19.5)	11.4	< 0.0001
**Sex**				
Male	1380 (38.0)	128 (19.5)	9.3	
Female	2251 (62.0)	527 (80.5)	23.4	< 0.0001
**Area of residence (Italy)**				
North	2490 (67.4)	545 (82.6)	21.9	
Centre	392 (10.6)	65 (9.8)	16.6	
South/islands	810 (21.9)	50 (7.6)	6.2	< 0.0001
**Education, years**				
≤ 8	447 (15.0)	51 (9.2)	11.4	
9–13	1532 (51.5)	299 (54.0)	19.5	
> 13	997 (33.5)	204 (36.8)	20.5	< 0.0001
**Smoking status**				
Never	2506 (70.6)	413 (65.5)	16.5	
Current	775 (21.8)	153 (24.3)	19.7	
Former	269 (7.6)	65 (10.3)	24.2	0.002
**Hair colour**				
Black	240 (6.6)	22 (3.4)	9.2	
Dark brown	1699 (47.0)	301 (46.2)	17.7	
Light brown	1265 (35.0)	245 (37.6)	19.4	
Blond/red	413 (11.4)	83 (12.8)	20.1	0.001
**Eye colour**				
Black/dark brown	1425 (39.5)	230 (35.5)	16.1	
Light brown	1049 (29.1)	202 (31.2)	19.3	
Green/blue	1130 (31.4)	216 (33.3)	19.1	0.067
**Phototype**				
I–II	690 (22.0)	100 (18.4)	14.5	
III–VI	2452 (78.0)	444 (81.6)	18.1	0.027
**Nevi, total number**				
≤ 10	568 (18.8)	50 (8.9)	8.8	
11–30	1109 (36.8)	169 (29.9)	15.2	
31–50	842 (27.9)	191 (33.8)	22.7	
51–100	322 (10.7)	95 (16.8)	29.5	
≥ 101	175 (5.8)	60 (10.6)	34.3	< 0.001
**Presence of freckles**				
No	2049 (78.2)	424 (79.3)	20.7	
Yes	571 (21.8)	111 (20.7)	19.4	0.289
**Presence of solar lentigines**				
No	1172 (44.8)	225 (41.7)	19.2	
Yes	1446 (55.2)	314 (58.3)	21.7	0.113
**Presence of actinic keratosis**				
No	2401 (93.7)	513 (97.0)	21.4	
Yes	161 (6.3)	16 (3.0)	9.9	0.001
**Use of sunbeds before 15 ys**				
No	3638 (98.5)	638 (96.7)	17.5	
Yes	54 (1.5)	22 (3.3)	40.7	< 0.0001
**History of sunburns before 15 ys**				
No	1798 (51.2)	275 (42.8)	15.3	
Yes	1716 (48.8)	367 (57.2)	21.4	< 0.0001
**History of sunburns after 15 ys**				
No	1688 (49.0)	200 (31.6)	11.8	
Yes	1757 (51.0)	433 (68.4)	24.6	< 0.0001
**Use of sunscreens**				
No	613 (16.8)	46 (7.0)	7.5	
Yes	3025 (83.2)	608 (93.0)	20.1	< 0.0001
**Subject “at risk”**^**d**^				
no	1489 (40.4)	172 (26.1)	11.6	
yes	2193 (59.6)	487 (73.9)	22.2	< 0.0001
*Risk factors*				
Common nevi > 40	943 (28.1)	256 (43.7)	27.1	< 0.0001
Presence of atypical nevi	452 (13.6)	76 (13.2)	16.8	0.764
1^st^ degree family history of skin cancer	276 (8.3)	51 (8.8)	18.5	0.596
Personal history of skin cancer	110 (3.3)	21 (3.6)	19.1	0.616
Presence of solar damage	903 (25.4)	227 (36.1)	25.1	< 0.0001
**One or more atypical lesion**				
No	3412 (92.7)	602 (91.4)	17.6	
Yes	270 (7.3)	57 (8.6)	21.9	0.152
**One or more suspicious lesion to be removed**				
No	3443 (93.5)	611 (92.7)	17.7	
Yes	239 (6.5)	48 (7.3)	20.1	0.362

*SD* standard deviation; *IQR*, interquartile range.

^a^Totals may vary because of missing values.

^b^χ^2^ test.

^c^Mann–Whitney test.

^d^Subjects were defined “at risk” if at least one condition was present among > 40 common nevi, atypical nevi, first degree family history of melanoma or non-melanoma skin cancer, personal history of skin cancer, presence of outcomes from solar damage (i.e. sunburns, solar lentigo etc.)

Variables entered into the multivariable model with use of sunbeds after the age of 15 years as outcome were: sex, age, area of residence, education, phototype, number of common nevi in the whole body, use of sunbeds before the age of 15, history of sunburns before age of 15, use of sunscreen, smoking, and to be a subject at risk for the presence of outcomes from solar damage. The multivariate analysis (Table 2) confirmed the associations found in the univariate analysis concerning female sex, younger age (< 35 years), higher educational level, geographic area, darker phototype, higher number of common nevi (p-trend < 0.0001), use of sunbeds before the age of 15 years, history of sunburns after the age of 15, use of sunscreens, smoking, and to be a subject at risk for the presence of outcomes from solar damage. In the complete-case analysis on 1687 individuals with full information on all variables in the model, female sex, age < 35 years, geographic area, darker phototype, number of common nevi (> 30), history of sunburns after the age of 15, smoking, and to be a subject at risk for the presence of outcomes from solar damage remained statistically significant.

**Table 2:** Results of the multivariable analysis with “use of sunbeds after 15 years of age” as dependent variable.

	OR^a^ (95% CI)	*P*value	*P*trend
**Sex**			
Male	1		
Female	3.52 (2.81–4.42)	< 0.0001	
**Age**			
≥ 55.0	1		
35.0–54.9	1.33 (0.99–1.78)	0.056	
< 35.0	2.68 (2.10–3.43)	< 0.0001	0.048
**Area of residence (Italy)**			
North	1		
Centre	0.64 (0.47–0.89)	0.007	
South/islands	0.28 (0.20–0.41)	< 0.0001	
**Education, years**			
≤ 8	1		
> 8	1.68 (1.19–2.36)	0.003	
**Phototype**			
I-II	1		
III-VI	1.56 (1.20–2.03)	0.001	
**Nevi, total number**			
≤ 10	1		
11–30	1.51 (1.05–2.16)	0.025	
33–50	1.92 (1.34–2.76)	< 0.0001	
51–100	2.43 (1.61–3.68)	< 0.0001	
≥ 100	2.87 (1.80–4.57)	< 0.0001	< 0.0001
**Use of sunbeds before 15 ys**			
No	1		
Yes	2.02 (1.09–3.73)	0.024	
**History of sunburns before 15 ys**			
No	1		
Yes	1.74 (1.42–2.13)	< 0.0001	
**Use of sunscreens**			
No	1		
Yes	1.48 (1.05–2.08)	0.026	
**Smoking**			
Never	1		
Current	1.59 (1.26–2.00)	< 0.0001	
Former	2.33 (1.65–3.30)	< 0.0001	
**Subject at risk for presence of solar damage**			
No	1		
Yes	1.66 (1.34–2.06)	< 0.0001	

*OR* odds ratio; *CI* confidence interval.

^a^OR adjusted for all items in table.

The prevalence of sunbeds use after the age of 15 years was negatively correlated with the lowest annual mean value of the maximum temperatures (Spearman’s ρ =  − 0.53, p = 0.029).

## Discussion

In this study, we observed that the prevalence of sunbed users after the age of 15 years in a group of voluntary participants in an Italian skin cancer prevention program was 22.2%, and 2.2% before the age of 15 years. In the Euromelanoma study on 30 European countries^9^ the overall prevalence of sunbed use was 10.6%, however, there were large differences among countries. Similar to our study, that survey estimated for Italy a 20.6% prevalence of use of tanning devices. In a previous report of the Italian Euromelanoma Day^10^, ever-users of solarium were 23.3% of the screened population, and another survey observed a prevalence of 20% in an area on the sunny Mediterranean coast in Italy^11^. The prevalence observed in all these studies is lower than that found in a previous meta-analysis on a group of Northern and Western European countries (42%)^6^. Such differences may be in part explained by the fact that people participating in a skin cancer campaign are generally more health-conscious than the general population. Moreover, the study by Stanganelli et al^11^ selected people living in sunny areas, and in the present study we observed that prevalence of sunbed users was significantly higher in Northern Italian regions compared to the Center and South of Italy.

In general, a positive correlation between indoor tanning and latitude has been reported^12^, even though in the European study^9^ Italy and Spain represented an exception with a frequent sunbed use despite being sunny countries.

The higher prevalence of indoor tanning in women compared to men is well-known^1,13^ and was confirmed by our study, with an odds ratio of 3.5. This has certainly socio-cultural and aesthetical reasons, but may be due also to the fact that men more often have outdoor activities and thus less need for artificial tanning. Interestingly, this difference concerns all European countries, though with different women to men ratios^9^. Also the observation that sunbed users were younger than non-users is in line with previous studies^13–15^. Besides cultural reasons, this may depend on the higher utilization of indoor tanning in recent years compared to the past. Another characteristic of sunbed users was the higher educational level, compared to non-users, as reported in previous studies^10,16,17^, likely for economic reasons. Then, sunbed use was more frequent in participants with darker skin types^13,17,18^. This result was not always reported in previous studies, where a high percentage of people with fair phototype used indoor tanning^11,19^. Indeed, also in our study the difference among phototypes was not striking, and also a high percentage of people with fair hair and fair skin used sunbeds. It is particularly worrying that people at higher risk for different factors, such as fair phototype, number of nevi, and solar damage, were more frequent users than people at lower risk.

The negative association between use of sunbeds and actinic keratosis was observed only in the univariate analysis, but it was not confirmed at multivariate level as it is due to the confounding effect of sex and age. In fact, the prevalence of AK was higher in older people and in males, while sunbed users were mostly young people and females. Our results confirm that it is still necessary to disseminate correct information on the risks associated with the use of indoor tanning, and that awareness campaigns addressed to the general public and to policy-makers are needed, as indoor exposure to UV rays represents a modifiable risk factor. In 2003, the World Health Organization (WHO) published a guidance document on sunbed legislation^20^, and in 2017 a booklet to inform policy-makers on health risks associated with artificial tanning^21^. The WHO recommended to avoid the use of sunbeds for people under the age of 18 years and with phototypes I and II. However, not all countries have adopted these recommendations. Causing an increased risk of skin cancer, indoor tanning is an important public health issue and a relevant aspect of primary prevention. Young women are particularly susceptible to the cult of tanning, thus increasing cancer risk and therefore affecting their health status throughout their life.

Our study concerned only the Italian population. This may seem a constraint, but, considering the importance of the above-mentioned factors in influencing lifestyle in different countries, a single-country study appears useful to elaborate specific primary prevention programs. On the other hand, the setting of our project was different from that of most other available studies, as it surveyed individuals who voluntarily accessed the services provided by a non-governmental organization to screen for skin cancer. That in such more health-concerned population we have observed practically the same levels of artificial tanning seen in hospitals and academic institutions is particularly worrisome.

A limitation of the study is that we do not have any information on people who refused to participate in the survey.

A deeper insight into the topic may contribute to identify the best prevention strategies at national level, which can also be an inspiration for countries that share similar cultural and climatic conditions as ours.

## Data Availability

The datasets generated and analysed during the current study are available from the corresponding author on reasonable request.
